# Microglial activation and blood–brain barrier permeability in cerebral small vessel disease

**DOI:** 10.1093/brain/awab003

**Published:** 2021-05-17

**Authors:** Jessica Walsh, Dan J Tozer, Hasan Sari, Young T Hong, Anna Drazyk, Guy Williams, N Jon Shah, John T O’Brien, Franklin I Aigbirhio, Gary Rosenberg, Tim D Fryer, Hugh S Markus

**Affiliations:** 1 Department of Clinical Neurosciences, University of Cambridge, Cambridge, UK; 2 Institute of Neuroscience and Medicine, Forschungszentrum Jülich, Jülich, Germany; 3 JARA–BRAIN–Translational Medicine, Aachen, and Department of Neurology, RWTH Aachen University, Aachen, Germany; 4 Department of Psychiatry, University of Cambridge, Cambridge, UK; 5 UNM Health Sciences Center, University of New Mexico, Albuquerque, NM 87106, USA

**Keywords:** cerebral small vessel disease, neuroinflammation, blood–brain barrier, lacunar stroke, CADASIL

## Abstract

Cerebral small vessel disease (SVD) is a major cause of stroke and dementia. The underlying pathogenesis is poorly understood, but both neuroinflammation and increased blood–brain barrier permeability have been hypothesized to play a role, and preclinical studies suggest the two processes may be linked. We used PET magnetic resonance to simultaneously measure microglial activation using the translocator protein radioligand ^11^C-PK11195, and blood–brain barrier permeability using dynamic contrast enhanced MRI. A case control design was used with two disease groups with sporadic SVD (*n = *20), monogenic SVD (cerebral autosomal dominant arteriopathy with subcortical infarcts and leukoencephalopathy, CADASIL), and normal controls (*n = *20) were studied. Hotspots of increased glial activation and blood–brain barrier permeability were identified as values greater than the 95th percentile of the distribution in controls. In sporadic SVD there was an increase in the volume of hotspots of both ^11^C-PK11195 binding (*P = *0.003) and blood–brain barrier permeability (*P = *0.007) in the normal appearing white matter, in addition to increased mean blood–brain barrier permeability (*P < *0.001). In CADASIL no increase in blood–brain barrier permeability was detected; there was a non-significant trend to increased ^11^C-PK11195 binding (*P = *0.073). Hotspots of ^11^C-PK11195 binding and blood–brain barrier permeability were not spatially related. A panel of 93 blood biomarkers relating to cardiovascular disease, inflammation and endothelial activation were measured in each participant; principal component analysis was performed and the first component related to blood–brain barrier permeability and microglial activation. Within the sporadic SVD group both hotspot and mean volume blood–brain barrier permeability values in the normal appearing white matter were associated with dimension 1 (β  =  0.829, *P = *0.017, and β  =  0.976, *P = *0.003, respectively). There was no association with ^11^C-PK11195 binding. No associations with blood markers were found in the CADASIL group. In conclusion, in sporadic SVD both microglial activation and increased blood–brain barrier permeability occur, but these are spatially distinct processes. No evidence of increased blood–brain barrier permeability was found in CADASIL.

## Teaser

Using advanced brain imaging techniques, Walsh *et al*. provide evidence of both neuroinflammation and increased blood-brain barrier permeability in patients with cerebral small vessel disease. Both processes could serve as potential therapeutic targets.

See Edison (doi:10.1093/brain/awab149) for a scientific commentary on this article.

## Introduction

Cerebral small vessel disease (SVD) causes a quarter of all strokes and is the major pathology underlying vascular dementia and vascular cognitive impairment.^1^ Disease of the small perforating arteries supplying the white matter and deep grey matter nuclei results in a number of characteristic pathologies well visualized on MRI including lacunar infarcts, white matter hyperintensities (WMH), cerebral microbleeds, and enlarged perivascular spaces.^2^ Both lacunes and WMH have been shown to predict stroke and dementia risk^3^. More advanced imaging techniques, particularly diffusion tensor imaging (DTI), show widespread abnormalities in the white matter, including outside WMH in the normal-appearing white matter (NAWM).^4^ These have been shown to correlate with the degree of cognitive impairment,^5^^,^^6^ and predict future dementia risk. ^7^

Despite its public health importance there are few proven treatments for SVD. Hypertension is a major risk factor, and anti-hypertensive treatment has been shown to reduce WMH progression and may reduce vascular cognitive impairment.^8^ However, beyond risk factor reduction, the absence of other therapeutic approaches for SVD reflects a lack of understanding of underlying pathophysiology, which is essential to inform the development of novel treatment approaches.

Two additional pathological processes that have been implicated in SVD pathogenesis are increased blood–brain barrier (BBB) permeability^9^ and neuroinflammation.^10^ Both neuropathological and CSF studies suggest increased leakage of plasma proteins across the BBB in SVD.^11^^,^^12^ Dynamic contrast enhanced (DCE) MRI studies have shown changes consistent with increased BBB permeability in patients with different manifestations of SVD including lacunar strokes, WMH, and vascular cognitive impairment, including in the NAWM.^13^^-^^15^ Following intravenous injection of a gadolinium contrast agent, changes in signal intensity can be found consistent with leakage of the contrast agent across the BBB.

Most studies have looked at BBB permeability across large regions such as the white matter, but the use of signal processing techniques such as Kalman filtering and Patlak modelling, allows calculation of maps showing the spatial disruption of BBB leakage in individual subjects. Using this technique focal regions or ‘hotspots’ of increased permeability in the white matter of patients with SVD have been described.^16^^,^^17^

Neuroinflammation has also been indicated in the pathogenesis of SVD. Support for this is provided by animal models,^18^ and neuropathological studies showing elevation of inflammatory markers in close proximity to diseased arteries.^19^^,^^20^ Circulating biomarkers of both inflammation and endothelial activation are increased in SVD, and in some studies these have been shown to predict WMH progression.^21^^,^^22^ However, clinical studies in humans have measured systemic inflammatory processes and there is little *in vivo* data supporting the role of inflammation in the disease. Neuroinflammation can been measured using PET imaging of radioligands for the 18 kDa translocator protein (TSPO), whose expression is upregulated in activated microglia. One such TSPO radiotracer ^11^C-PK11195 has demonstrated increased ligand binding in neurodegenerative diseases including Alzheimer’s disease,^23^^,^^24^ but there is little data for TSPO PET in SVD.

A pathophysiological cascade linking BBB disruption and inflammation has been proposed in an animal model of white matter ischaemia. Chronic inflammation resulted in white matter hypoperfusion and hypoxia, which led to an increase in HIF1A inducing an inflammatory response, with release of matrix metalloproteinases (MMPs) that disrupted the extracellular matrix of the vascular endothelium leading to opening of the BBB.^18^ This hypothesis implies that increases in BBB permeability and microglial activation may be related.

In this study we acquired both DCE-MRI data to estimate BBB permeability, and also ^11^C-PK11195 PET data to estimate microglial activation. We determined whether there was evidence of increased BBB permeability, and particularly hotspots of increased permeability, and how these related to WMH. We further determined whether there were regions of increased microglial activation, and if so, how these related spatially to regions of increased BBB permeability. To determine whether these processes were relevant across different types of SVD we applied the technique to patients with sporadic SVD, for which the major risk factor is hypertension, as well as those with the monogenic form of SVD, cerebral autosomal dominant arteriopathy with subcortical infarcts and leukoencephalopathy (CADASIL), and healthy controls.

## Materials and methods

### Participants

Three groups of participants were studied, two with SVD (sporadic SVD, *n *=* *20; monogenic SVD CADASIL, *n *=* *20) and one control group (*n *=* *20).

Twenty patients with sporadic SVD were included. Inclusion criteria were clinical evidence of lacunar stroke syndrome, with a corresponding lacunar infarct on diffusion-weighted imaging at the time of stroke for cases initially imaged within 3 weeks of stroke or an anatomically compatible lacunar infarct (≤1.5 cm diameter) on FLAIR/T_1_ MRI for cases imaged later after stroke, and in addition WMH rating of ≥2 on the Fazekas scale.^25^ Any cause of stroke other than SVD (e.g. large artery stenosis >50% or cardioembolic cause), or a cortical infarct were exclusion criteria. Participants were recruited from an inpatient stroke service and outpatient stroke clinic.

The inclusion criteria for 20 patients with monogenic SVD CADASIL were a confirmed genetic diagnosis of CADASIL, as defined by a typical cysteine changing *NOTCH3* mutation, and age >18 years. Participants were recruited from a national CADASIL clinic based at Cambridge.

Participants in both SVD groups were not recruited until at least 3 months after a stroke to avoid changes secondary to acute injury, and in both groups MMSE had to be >21 to exclude those with more severe cognitive impairment and ensure subjects could comply with the protocol.

The healthy control group comprised 20 participants with no history of stroke or other major neurological disorder and were recruited from both the community and family/friends of patients.

Further exclusion criteria in all three groups were: contraindication to MRI, females of childbearing potential and/or who were breastfeeding due to the administration of the radioactive ligand, and an eGFR (estimated glomerular filtration rate) ≥ 59 ml/min/1.73 m^2^ in view of the gadolinium contrast administration.

#### Approvals

The study was approved by the East of England—Cambridge South Ethics Committee (REC no: 16/EE/0468, IRAS project ID: 212632), and the Administration of Radioactive Substances Advisory Committee (ARSAC ref: 83/3886/35752). All participants provided written informed consent.

### PET MRI acquisition

All participants underwent PET and MRI in a single session on a GE SIGNA PET/MR scanner (GE Healthcare) at the Wolfson Brain Imaging Centre in Cambridge, UK. The scanner can simultaneously acquire PET and 3 T MRI data. A 32-channel NOVA head coil (Nova Medical) was used for MRI data acquisition. ^11^C-PK11195 produced at the Wolfson Brain Imaging Centre Radiopharmaceutical Unit was injected over ∼30 s and list-mode PET data were acquired for 75 min. The median injected activity was 440 MBq [interquartile range (IQR) 401–483 MBq] with corresponding injected mass values of 3.9 (IQR 2.8–6.4) μg.

Simultaneously, whole brain non-contrast MRI was acquired. Non-contrast MRI sequences included: (i) 3D T_1_-weighted fast-spoiled gradient echo sequence (BRAVO), flip angle = 12°, inversion time = 450 ms, field of view =28 mm, slice thickness = 1 mm, number of slices = 192, reconstructed matrix size = 512 × 512; (ii) axial susceptibility weighted imaging, flip angle = 17°, repetition time = 40.6 ms, echo time = 24.2 ms, field of view = 22 mm, slice thickness = 2 mm, number of slices = 70, reconstructed matrix size = 256 × 256; (iii) axial T_2_ FLAIR sequence, angled anterior commissure-posterior commissure (AC-PC), flip angle = 160°, repetition time = 8800 ms, echo time = 120 ms, inversion time = 2445 ms, field of view = 22 mm, slice thickness = 5 mm, number of slices = 28, reconstructed matrix size = 256 × 256; and (iv) axial T_2_ fast spoiled gradient echo sequence in AC-PC angle, flip angle = 111°, echo time = 85 ms, repetition time = 6000 ms, field of view = 22 mm, slice thickness = 5 mm, number of slices = 31, reconstructed matrix size = 1024 × 1024.

Following the non-contrast MRI, DCE-MRI was acquired in a subsection of the brain chosen by the radiographer to include the regions of characteristic SVD damage. Gadolinium [gadoterate meglumine (Dotarem^®^)] was injected intravenously at a dose of 0.025 mmol/kg at an injection rate of 6 ml/s. T_1_ was mapped prior to injection, followed by ∼22 min of T_1_ mapping post-injection.

The T_1_ mapping sequence uses a 3D radiofrequency (RF)-spoiled gradient echo imaging sequence to obtain T_1_ relaxation times. Six flip angles were used (2°, 5°, 12°, 17°, 22°, 27°) to calculate each map. Eight post injection maps with a temporal resolution of ∼2.5 min were collected. Imaging parameters were: repetition time = 6.3 ms, echo time = 1.784 ms, resolution = 2 × 2 × 3 mm (reconstructed to 0.94 × 0.94 × 3 mm), number of slices = 16 and reconstructed matrix size = 256 × 256. In addition, a B0 mapping sequence for flip angle correction was acquired prior to injection; flip angle = 15°, number of echoes = 1, receiver bandwidth = 15.63, field of view = 35 mm and slice thickness = 5 mm.

### Non-contrast magnetic resonance analysis

WMH were quantified on FLAIR images by a single trained rater using the semi-automatic contouring technique Jim analysis software version 7.0.5 (Xinapse Systems Limited, http://www.xinapse.com/j-im-7-software/). Whole brain WMH maps were generated. To assess reproducibility 10 FLAIR scans were remarked in a randomized, blinded setting, by a second experienced rater and were used to create inter-rater and intra-rater reliability coefficients. The inter-rater and intra-rater reliability coefficients were 0.988 and 0.993, respectively.

Lacunes were defined as CSF-filled cavities at least 3 mm in diameter.^2^ They were manually delineated on FLAIR images by a single neurologist, blinded to subject identity, with both T_1_ and FLAIR scans being visually inspected to confirm the presence of lacunes. The FLAIR image was registered to the T_1_ image using a rigid body transformation in Advanced Normalization Tools (ANTs, http://stnava.github.io/ANTs/). The resulting transformation was used to resample the WMH mask from the FLAIR image to the T_1_ image using nearest-neighbour interpolation.

Each T_1_ BRAVO image was processed using the ‘segment’ routine in SPM12 (https://www.fil.ion.ucl.ac.uk/spm/software/spm12/). SPM segmentation provides tissue probability maps and volumes for each tissue class were calculated as the sum of voxels that have a probability of >0.5 of belonging to that class, after removal of voxels in the WMH mask. The tissue segments and WMH mask were used to create the NAWM and white matter (sum of NAWM and WMH) masks. The masks were then eroded by 3 mm using fslmaths (https://fsl.fmrib.ox.ac.uk/fsl/fslwiki), to effectively eliminate ventricular or grey matter contamination.

### DCE-MRI analysis

Permeability maps were created using previously published methods.^16^ The T_1_ maps from the DCE-MRI are used to calculate estimates of gadolinium concentration in tissue, assuming a linear relationship. Patlak graphical analysis^26^ was then applied to the gadolinium concentration images over time to determine influx rate (K_i_) as a metric of permeability. This technique requires an arterial input function, representing the concentration of gadolinium in arterial plasma over the course of the scan. There is no artery in the field of view of our study and therefore the sagittal sinus was used, corrected by the factor (1 − haematocrit), and assumed to be representative of the arterial concentration of contrast agent.^16^

Global values of BBB permeability in the white matter, NAWM and WMH masks were calculated as the mean K_i_ values across the voxels in that region. Hotspots were classified as voxels with a K_i_ value greater than the 95th percentile of the K_i_ distribution in NAWM of the control population. The tissue segments and K_i_ maps were then used along with this threshold to generate hotspot maps. The volume of these hotspots in white matter, NAWM and WMH was then determined.

To determine whether BBB permeability and ^11^C-PK11195 binding differed in the NAWM in close proximity to the WMH compared to the rest of the NAWM, a ‘penumbra’ around the WMH was delineated. This was defined by taking the WMH mask in T_1_ mapping space and dilating this by 3 mm in all directions. The WMH mask was then subtracted from the dilated mask to give the penumbra. This was further masked using the CSF and grey matter segments to leave only white matter. Again, global and hotspot parameter values were obtained in the penumbra.

### PET analysis

List-mode PET data were histogrammed into 55 time frames and then reconstructed into images (128 × 128 × 89 matrix; 2.0 × 2.0 × 2.8 mm voxel size) using time-of-flight ordered subsets expectation maximization^27^ with 16 subsets, six iterations and no smoothing. Attenuation correction included the use of a multi-subject atlas method^28^ and improvements to the MRI brain coil component. Image reconstruction also included corrections for random coincidences, dead time, normalization, scattered coincidences, radioactive decay, and sensitivity.

SPM12 (https://www.fil.ion.ucl.ac.uk/spm/software/spm12/) was used to realign each dynamic image series and hence ameliorate the impact of head motion. A mean realigned PET image was then used to co-register each realigned dynamic PET image series to the T_1_ BRAVO magnetic resonance image from the same scan.

To estimate specific binding of ^11^C-PK11195, binding potential relative to a non-displaceable compartment (BP_ND_) was determined with a basis function version of the simplified reference tissue model (SRTM) incorporating correction for vascular binding.^29^ The white matter reference tissue input was estimated with supervised cluster analysis^30^ using library data determined from control scans of another ^11^C-PK11195 project on the same scanner with the same acquisition and processing protocol. A 4 mm full-width at half-maximum Gaussian was applied to the dynamic images prior to production of BP_ND_ maps using SRTM. Within the co-registered NAWM and WMH masks, mean BP_ND_ was determined and BP_ND_ hotspots were classified in the same way as for BBB permeability, using the 95th percentile of the BP_ND_ distribution in the control population. Penumbra values were calculated in the same way as documented above for BBB permeability.

TSPO, is expressed by cell types other than microglia, including endothelial cells. To account for this, correction of the PET signal for binding to vascular endothelial cells has been developed^29^ and this was applied in the reference tissue kinetic model used in this work to increase the specificity for TSPO expressed in parenchymal brain tissue.

### Blood biomarkers

Blood was taken from each participant at the same time as the PET/MR scan, centrifuged, and the serum was separated and stored at −70°C. A panel of 93 blood biomarkers relating to cardiovascular disease, inflammation and endothelial activation were measured in each participant. These comprised 92 markers from the Olink Proteomics Cardiovascular Disease III panel (https://www.olink.com/products/cvd-iii-panel/), together with measurement of C-reactive protein (CRP) using the Siemens Dimension high sensitivity CRP assay at the Core Biochemical Assay Laboratory, Addenbrooke’s Hospital, Cambridge, UK. All assays were performed at the same time to avoid batch effect and blinded to subject identity.

### Statistical analysis

All analyses were carried out in R statistics (version 3.3.1 2016, R Foundation for Statistical Computing, Vienna, Austria).

The demographic data consisted of frequency and continuous data. For frequency data, chi-squared tests were used, while for continuous data *t*-tests were used. WMH volume and lesion number were not normally distributed, so the tests were performed on transformed variables (cube root).

Group differences for BBB permeability (K_i_), ^11^C-PK11195 binding (BP_ND_) and other MRI markers were carried out using ANCOVA tests controlled for age and sex, with Bonferroni adjustment for multiple comparisons. If a volumetric measure was used (WMH volume or lacune volume), then the analyses were additionally controlled for intracranial volume. For these analyses distributions for all variables were examined for normality and the following transformations performed to obtain normal distributions: BBB permeability, WMH volume, lacune volume, and lacune count were all transformed with cube root; and ^11^C-PK11195 binding was log-transformed.

Chi-squared tests were used to assess the independence of BBB permeability and ^11^C-PK11195 binding on a voxel-by-voxel basis for all regions for all groups, except for the WMH of the control group. Voxels were divided into four groups depending on whether the voxel K_i_ and BP_ND_ values were above the corresponding 95th percentile thresholds: (i) K_i_ only; (ii) BP_ND_ only; (iii) both K_i_ and BP_ND_; or (iv) neither K_i_ nor BP_ND_. The WMH of the control group had an expected frequency of <5 in one of the cells, and therefore did not meet the assumptions of the chi-squared test; a Fisher’s exact test was used instead.

To test whether hotspots of BBB permeability or ^11^C-PK11195 binding were more likely to co-localize with WMH comparisons between the percentage volume of hotspots in NAWM and WMH were performed using Wilcoxon signed rank tests for paired non-parametric data. These tests were performed on the raw untransformed values. *P*-values were corrected for multiple comparisons using the false discovery rate correction.

Regional comparisons between the BBB permeability and ^11^C-PK11195 binding in the penumbra compared to NAWM were carried out with Wilcoxon signed rank tests for paired non-parametric data. These tests were performed on the raw untransformed values and *P*-values were then adjusted using Bonferroni correction.

To compare the blood biomarkers with the imaging parameters a single blood biomarker variable was calculated using principal component analysis (PCA). PCA was performed on the 93 biomarkers for the sporadic SVD and CADASIL groups individually, and the coordinates for dimension 1 were extracted to be investigated in association with BBB permeability and ^11^C-PK11195 binding using multivariate linear models controlled for age and sex. Dimension 1 was different for each of the groups, as PCA was performed on each of the groups separately.

Multivariate linear models, controlled for age and sex, were used to investigate if other imaging markers were significantly associated with BBB permeability or ^11^C-PK11195 binding. If a volumetric measure was used (WMH volume or lacune volume), then the analyses were additionally controlled for intracranial volume. *P*-values were adjusted for multiple comparisons using the Benjamini–Hochberg procedure.

### Data availability

The data that support the findings of this study are available from the corresponding author, upon reasonable request.

## Results

### Participants

Twenty control, 20 sporadic SVD and 20 CADASIL subjects were recruited. DCE-MRI data were available for 19 in each group; the three failures were due to: a scanner problem, a gadolinium injection failure, and excess motion for one subject. PET data were acquired for 17 control, 16 sporadic SVD and 14 CADASIL participants; the scans that did not take place were due to radiotracer production or quality control issues. Demographics and clinical details from the three groups are shown in Table 1. Cognitive data in the three groups are provided in Supplementary Table 1.

**Table 1:** Demographics, medical history, conventional MRI markers of SVD and PET injection details in the three groups

Variable	Group			*P*-value	
	Control	Sporadic SVD	CADASIL	SVD versus control	CADASIL versus control
	*n = *20	*n = *20	*n = *20		
Age, years	66.4 (6.7)	70.9 (9.0)	51.9 (9.5)	0.08	**<0.001**
Male sex	13 (65)	10 (50)	13 (65)	0.52	1.00
Ethnicity: Caucasian	20 (100)	20 (100)	18 (90)	–	–
Education, years	13.4 (3.8)	13.0 (2.9)	14.1 (3.3)	0.68	0.54
BMI, kg/m^2^	26.3 (3.3)	27.9 (5.9)	28.8 (4.5)	0.34	0.08
Hypertension	6 (30)	18 (90)	8 (40)	**<0.001**	0.74
Hyperlipidaemia	7 (35)	11 (55)	3 (15)	0.34	0.27
Diabetes	1 (5)	4 (20)	0 (0)	0.34	1.00
Smoking	12 (60)	11 (55)	7 (35)	1.00	0.21
Past history of stroke	0 (0)	20 (100)	8 (40)	**<0.001**	**0.006**
White matter hyperintensity volume, cm^3^	3.1 (6.0)	26.6 (26.0)	61.1 (40.2)	**<0.001**	**<0.001**
Number of lacunes	0.05 (0.22)	1.70 (1.72)	5.20 (7.19)	**<0.001**	**<0.001**
Injected PK11195 mass, μg; median not mean as non-normal	4.4 (4.0)	4.2 (2.7)	3.5 (3.3)	0.90	0.36
Injected ^11^C-PK11195 activity per unit body weight, MBq/kg	5.65 (1.15)	6.21 (2.29)	4.77 (1.63)	0.39	0.10

Values are presented as mean (SD) or *n* (%). Comparisons were made with the chi-square test for categorical data and the *t*-test for continuous data, with the exception of injected PK11195 mass which was compared using the Mann-Whitney test.

Compared with controls, age was not significantly different in sporadic SVD, but it was significantly lower in the CADASIL group (*P < *0.001). Hypertension was more common in SVD cases than controls (*P < *0.001). Group comparisons for other MRI parameters can be found in the Supplementary material.

### Blood–brain barrier permeability

**Figure 1:**
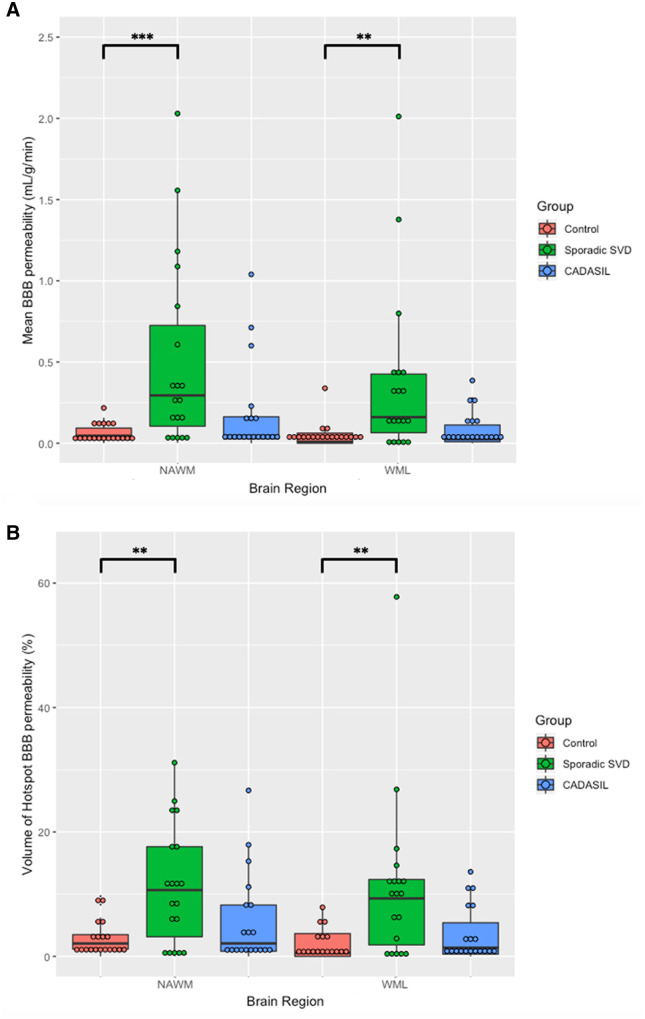
**BBB permeability comparisons between the groups.** (**A**) Mean BBB permeability (*K_i_*). Values were higher in sporadic SVD compared to control for NAWM (*P < *0.001) and WMH (WML) (*P = *0.003). Values were not significantly different between CADASIL and control in the NAWM (*P = *0.34) or WMH (*P = *0.51). (**B**) Volume of BBB permeability hotspots. Values were significantly higher in sporadic SVD compared to control in NAWM (*P = *0.007) and WMH (*P = *0.004). Values were not significantly different between CADASIL and control in the NAWM (*P = *0.87) or WMH (*P = *0.51). *P < 0.05, **P < 0.01, ***P < 0.001.

Mean white matter BBB permeability (K_i_) was significantly elevated in both the NAWM (*P < *0.001) and WMH (*P = *0.003) in the sporadic SVD group compared to controls (Fig. 1 and Table 2). Similarly, the volume of BBB permeability hotspots was significantly larger for the sporadic SVD group compared to controls in NAWM (*P = *0.007) and WMH (*P = *0.004).

**Table 2:** Mean value and hotspot volume for BBB permeability and ^11^C-PK11195 binding across the three groups

		Group			ANCOVA result	
		Control	Sporadic SVD	CADASIL	SVD versus control	CADASIL versus control
**BBB permeability**		***n = *19**	***n = *19**	***n = *19**		
Mean K_i_ (×10^−3^ ml/g/min)	NAWM	0.06 (0.06)	0.50 (0.58)	0.18 (0.29)	***F*(1,34) = 15.34** ***P *<* *0.001**	*F*(1,34) = 1.95 *P = *0.34
	WMH	0.04 (0.08)	0.38 (0.52)	0.08 (0.11)	***F*(1,32) = 12.07** ***P = *0.003**	*F*(1,32) = 1.34 *P = *0.51
Hotspot volume, %	NAWM	2.78 (2.75)	11.31 (9.50)	5.60 (7.40)	***F*(1,34) = 9.94** ***P = *0.007**	*F*(1,34) = 0.63 *P = *0.87
	WMH	1.94 (2.52)	11.14 (13.31)	3.52 (4.47)	***F*(1,32) = 11.59** ***P = *0.004**	*F*(1,32) = 1.35 *P = *0.51
**^11^C-PK11195 binding**		***n = *17**	***n = *16**	***n = *14**		
Mean BP_ND_	NAWM	0.04 (0.03)	0.07 (0.07)	0.06 (0.04)	*F*(1,29) = 1.14 *P = *0.59	*F*(1,27) = 1.05 *P = *0.63
	WMH	0.06 (0.12)	0.01 (0.09)	0.02 (0.05)	*F*(1,27) = 0.67 *P = *0.84	*F*(1,25) = 0.02 *P = *1.00
Hotspot volume, %	NAWM	4.77 (2.89)	11.02 (9.46)	11.28 (11.65)	***F*(1,29) = 12.48** ***P = *0.003**	*F*(1,27) = 4.86 *P = *0.07
	WMH	0.63 (1.31)	3.76 (5.95)	2.18 (2.10)	***F*(1,27) = 11.89** ***P = *0.004**	***F*(1,25) = 20.52** ***P *<* *0.001**

Values are presented as mean (SD). Significant* P*-values are shown in bold.

There was no significant difference in the mean value for BBB permeability (K_i_) or the volume of the BBB permeability hotspots between the CADASIL and control group in the NAWM or WMH (Table 2). In contrast to the sporadic SVD group, not all CADASIL patients had had a stroke; to determine the influence of this disparity, mean BBB permeability and the volume of hotspots were compared between those CADASIL cases with lacunes (*n *=* *11) versus those without (*n *=* *8), and no significant differences were found.

### ^11^C-PK11195 binding

The volume of hotspots of ^11^C-PK11195 binding was significantly higher in sporadic SVD compared to control in both the NAWM (*P = *0.003) and WMH (*P = *0.004). The volume of hotspots of ^11^C-PK11195 binding was not significantly different between CADASIL and control in NAWM, although there appeared to be a trend to higher values (*P = *0.073), but it was significantly higher in the CADASIL group compared to control in WMH (*P < *0.001).

**Figure 2:**
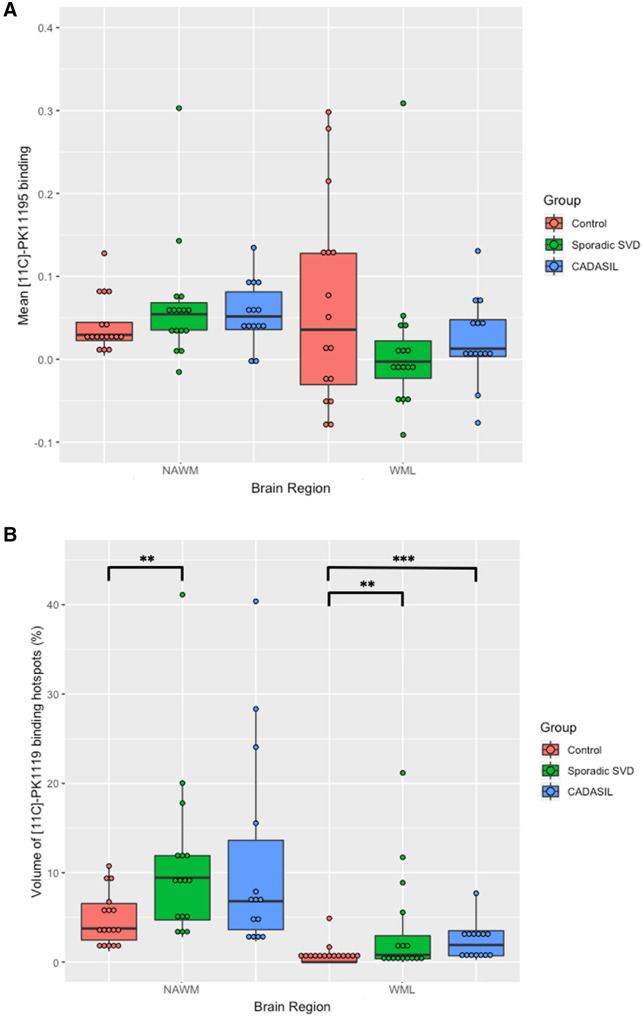
**^11^C-PK11195 binding comparisons between the groups.** (**A**) Mean ^11^C-PK11195 BP_ND_. Values were not different between sporadic SVD and control in the NAWM (*P = *0.59) or WMH (WML) (*P = *0.63). Similarly, values were not different between CADASIL and control in the NAWM (*P = *0.84) or WMH (*P = *1.00). (**B**) Volume of ^11^C-PK11195 binding hotspots. Values were significantly higher in the sporadic SVD group in the NAWM (*P = *0.003) and WMH (*P = *0.004). Values were not different between CADASIL and control in the NAWM (*P = *0.073) but were significantly higher in the CADASIL group in the WMH (*P < *0.001). *P < 0.05, **P < 0.01, ***P < 0.001.

There was no difference in mean NAWM ^11^C-PK11195 binding (BP_ND_) in sporadic SVD compared to control (*P = *0.589) or CADASIL compared to control (*P = *0.630) (Fig. 2). Further, there was no significant difference in mean WMH ^11^C-PK11195 BP_ND_ in sporadic SVD compared to control (*P = *0.844) or CADASIL compared to control (*P = *1.000).

### Spatial overlap analysis

**Figure 3:**
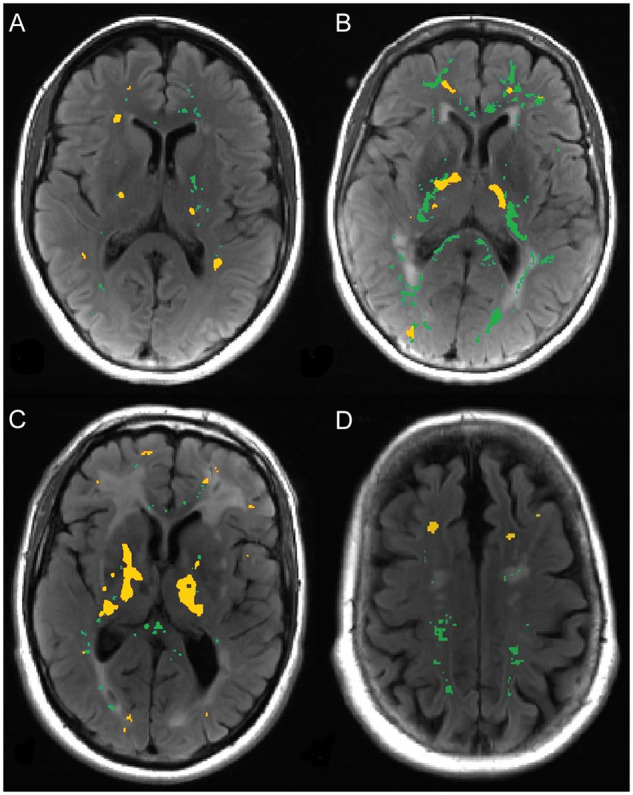
**Hotspots of increased BBB permeability and ^11^C-PK11195 binding.** Example images from four different SVD subjects (**A**–**D**) showing hotspots of increased BBB permeability (green) and ^11^C-PK11195 binding (yellow) overlaid onto T_2_ FLAIR images.

The spatial relationship between voxels of increased BBB permeability and ^11^C-PK11195 binding was investigated within the two patient groups (Fig. 3). In the sporadic SVD group, there were significantly fewer overlapping voxels than expected by chance in both NAWM (*P < *0.001) and WMH (*P < *0.001), indicating that the two processes did not co-localize spatially. In the CADASIL group, there were significantly fewer overlap regions than expected in NAWM (*P < *0.001), but WMH was similar to chance (*P = *0.21).

### Normal-appearing white matter versus white matter hyperintensities hotspot comparison

For all three subject groups there was a larger BBB permeability hotspot volume in NAWM compared to WMH (Table 3), similarly ^11^C-PK11195 binding hotspots were more prevalent in NAWM than WMH for all three groups (Table 3).

**Table 3:** Comparisons between hotspot volumes in NAWM and WMH for BBB permeability and ^11^C-PK11195 binding for the three subject groups

Group	Imaging parameter	NAWM		WMH		*P*-value
		Median	IQR	Median	IQR	
**BBB permeability**						
Control	Hotspot permeability volume, %	2.074	2.314	0.543	3.661	**0.024**
Sporadic SVD	Hotspot permeability volume, %	10.660	14.480	9.314	10.518	0.709
CADASIL	Hotspot permeability volume, %	2.088	7.429	1.393	5.027	**0.016**
**^11^C-PK11195 binding**						
Control	Hotspot PK binding volume, %	3.761	4.085	0.000	0.671	**<0.001**

Significant *P*-values are shown in bold.

### White matter hyperintensities penumbra

Mean BBB permeability and ^11^C-PK11195 binding in the penumbra were compared with the corresponding values in NAWM (Table 4). BBB permeability values were not different from those in the rest of the NAWM, whereas ^11^C-PK11195 binding in the penumbra was significantly lower than in the NAWM in the control, sporadic SVD and CADASIL groups (*P < *0.001 for all).

**Table 4:** Comparison of BBB permeability and ^11^C-PK11195 binding between NAWM and penumbra

Group	Median [IQR]		*P*-value
	NAWM	Penumbra
**BBB permeability (K_i_) (ml/g/min)**			
Control	0.046 [0.071]	0.045 [0.054]	0.85
Sporadic SVD	0.295 [0.620]	0.286 [0.534]	0.67
CADASIL	0.046 [0.136]	0.055 [0.163]	0.96
**^11^C-PK11195 binding (BP_ND_)**			
Control	0.030 [0.021]	−0.054 [0.041]	**<0.001**
Sporadic SVD	0.054 [0.033]	−0.090 [0.066]	**<0.001**
CADASIL	0.052 [0.045]	−0.084 [0.034]	**<0.001**

Significant *P*-values are shown in bold. Values are provided as median [IQR].

### Blood biomarkers

The first component derived from PCA on the 93 blood biomarkers explained a large amount of the variance (33.3% in sporadic SVD, 36.9% in CADASIL). Details on dimension 1 for each group can be found in the Supplementary material.

Within the SVD group, both mean and hotspot volume BBB permeability values were significantly associated with dimension 1 in NAWM (β  =  0.829, *P = *0.017, and β  =  0.976, *P = *0.003, respectively), and in WMH (β  =  0.737, *P = *0.030, and β  =  0.788, *P = *0.017, respectively) (Table 5). There was no association between dimension 1 and ^11^C-PK11195 binding. In the CADASIL group there was no association between dimension 1 and either BBB permeability or ^11^C-PK11195 binding.

**Table 5:** Associations between blood biomarkers and BBB permeability and ^11^C-PK11195 binding

Group	Measure	Region	β-Value	*P*-value
**Sporadic SVD**	**BBB permeability**
	Mean K_i_ (ml/g/min)	NAWM	0.829	0.016
		WMH	0.737	0.030
	Hotspot volume, %	NAWM	0.976	0.003
		WMH	0.788	0.016
	**^11^C-PK11195 binding**
	Mean BP_ND_	NAWM	−0.586	0.50
		WMH	−0.172	0.72
	Hotspot volume, %	NAWM	−0.298	0.69
		WMH	−0.555	0.50
**CADASIL**	**BBB permeability**
	Mean K_i_ (ml/g/min)	NAWM	0.138	0.94
		WMH	0.085	0.94
	Hotspot volume, %	NAWM	0.051	0.94
		WMH	0.021	0.94
	**^11^C-PK11195 binding**
	Mean BP_ND_	NAWM	−0.078	0.81
		WMH	0.224	0.80
	Hotspot volume, %	NAWM	0.168	0.80
		WHM	−0.268	0.80

Table shows associations between blood biomarkers and both BBB permeability and ^11^C-PK11195 binding using a linear model between dimension 1 of the blood biomarker PCA and both BBB permeability and ^11^C-PK11195 binding parameters. Significant *P*-values are shown in bold; all *P*-values are after FDR correction.

## Discussion

In this study, in which we acquired simultaneous estimates of BBB permeability and ^11^C-PK11195 binding (a marker of microglial activation), we demonstrated increased BBB permeability in sporadic SVD consistent with results of previous studies. In addition, we demonstrated focal ‘hotspots’ of both increased BBB permeability and increased microglial activation within the white matter in sporadic SVD. Regions of increased BBB permeability did not overlap with regions of increased microglial activation implying that they are spatially distinct processes. In contrast to sporadic SVD, in the monogenic form of SVD, CADASIL, we were unable to demonstrate any global increase, or hotspots, in BBB permeability. There were, however, foci of microglial activation, although these were only significantly increased in WMH. Taken together our data provide important new evidence that both increased BBB permeability and microglial activation may play a role in the pathogenesis of sporadic SVD, but that BBB permeability may be less important in CADASIL.

An increasing body of evidence implicates BBB permeability in the pathogenesis of sporadic SVD. Previous studies in patients with a variety of manifestations of SVD including WMH, lacunar infarcts, and vascular cognitive impairment have shown globally increased BBB permeability within the white matter,^13–15^ while one study has demonstrated the presence of hotspots of increased permeability using a similar modelling technique to that in our study.^13^

It has been suggested that endothelial dysfunction and activation may play an important role in disease pathogenesis. Abnormalities of the endothelium have been demonstrated post-mortem,^31^ while circulating markers of endothelial activation are increased in SVD^22^ and predict progression of WMH.^21^ To determine the relationship between blood markers of cardiovascular dysfunction including endothelial activation and inflammation we analysed 93 metabolites in blood relating to these processes. In patients with sporadic SVD a measure summarizing this data, the first principal component of a PCA analysis was associated with both increased white matter permeability, and volume of permeability hotspots, providing further evidence that these processes are important in pathogenesis.

Neuroinflammation has also been implicated in the pathogenesis of sporadic SVD, although there is limited direct data supporting this. Increased neuroinflammation has been shown in post-mortem tissue,^32^ altered innate immunity has been demonstrated and related to disease progression^33^ and, as described earlier, blood markers of endothelial activation and inflammation are increased in sporadic SVD. However, there have not been previous *in vivo* studies in humans, of which we aware, in which the presence of ‘neuroinflammation’ has been demonstrated. We demonstrated regions of increased microglial activation in white matter, both in NAWM and WMH, in patients with sporadic SVD implying that neuroinflammation is present in sporadic SVD, although our results do not determine whether it is merely associated or causally related. We did not detect a significant increase in mean white matter ligand uptake, but there was a trend towards increased uptake in the disease groups for the NAWM, and the lack of significance may have reflected a lack of power due to the modest sample size. A recent study has reported increased ^11^C-PK11195 binding associated with radiological SVD in patients with cognitive impairment.^34^ The power could have been further reduced by the fact that PET data were not available for six CADASIL and four sporadic SVD cases.

In an animal model of SVD a cascade of processes involving neuroinflammation, and BBB permeability has been demonstrated.^18^ This might imply that the two processes are spatially related. Using a combination of PET and MRI we were able to examine this hypothesis, but found no evidence of overlap between hotspots of microglial activation and BBB permeability. This suggests that the two processes are spatially distinct at a single individual time point, although given that our study was cross-sectional we cannot exclude the possibility that the two processes could occur in the same location at different temporal stages of the disease process. Furthermore, relationships between inflammation and BBB permeability may differ according to disease stage.^35^ Systemic inflammation was found to induce migration of brain resident microglia to the cerebral vasculature. These vessel-associated microglia were found to initially maintain BBB integrity but following sustained inflammation microglia phagocytosed astrocytic end-feet and impaired BBB function.

We also studied a group of patients with CADASIL. While the pattern of white matter damage on MRI is similar, the underlying molecular processes differ. CADASIL results from stereotyped mutations in the *NOTCH3* gene, which initiate a cascade of molecular events resulting in impaired vascular function.^36^ In a transgenic mouse model of CADASIL, no increase in BBB permeability was detectable,^37^ although a small study in humans found elevated CSF-serum albumin ratio suggesting leakage across the BBB.^38^ However, there is limited data examining this in humans. In contrast to sporadic SVD, we were unable to detect any increase in BBB permeability in the CADASIL group supporting the findings from the animal model, suggesting that the role of BBB permeability may differ between CADASIL and sporadic SVD. It is possible that BBB permeability could occur secondary to lacunar infarction, and in contrast to the sporadic SVD cases, not all CADASIL cases had suffered stroke. However, there were no differences in permeability measurements between those CADASIL cases with and without lacunes on MRI. Consistent with the lack of increased BBB permeability, there was no association between the blood markers of cardiovascular dysfunction and BBB permeability within the CADASIL group.

In contrast to the lack of increased BBB permeability seen in CADASIL, we did find some evidence of increased microglial activation in CADASIL although this only reached significance in the WMH.

A number of processes have been suggested to contribute to growth of WMH. These include both incorporation of new lacunar infarcts into confluent areas of WMH,^39^ as well as the gradual expansion of WMH with growth into a penumbra of ‘abnormal tissue’.^40^ We defined a penumbra around WMH as a 3-mm wide region surrounding the lesions and determined whether BBB permeability or microglial activation was altered in this region. We compared measurements with those in the rest of the NAWM. We found no increase in BBB permeability within the ‘penumbra’. There was a reduction in microglial activation rather than any increase within the penumbra. The explanation for this is uncertain.

Our study has a number of strengths. We studied well-characterized groups including both sporadic and genetic forms of SVD. We combined measurements of BBB permeability and ^11^C-PK11195 binding in the same patients and at the same time points using PET-magnetic resonance. Furthermore, these imaging analyses were combined with assays of circulating inflammatory blood biomarkers. Our study does also have limitations. The study was cross-sectional and we recruited only a moderate number of patients, and PET data were not available in a number of cases due to problems with radiopharmaceutical production. This reduced the power to detect associations, particularly in the CADASIL group. We included a single control group which was older than the CADASIL control group, although we controlled for age in all analyses.

In summary our results demonstrate that regions of increased BBB permeability and microglial activation both occur in sporadic SVD but are spatially distinct. It provides further evidence that these processes may represent therapeutic targets for the disease. However, because of the cross-sectional design of our study it demonstrates association but not causality, and longitudinal and intervention studies are required to determine whether these processes do contribute to progression of white matter damage and clinical symptoms. Our results suggest that different processes may be important in CADASIL, with increased BBB permeability playing a less important role, but we did find evidence of regions of increased microglial activation in both monogenic and sporadic forms of SVD.

## Supplementary Material

awab003_Supplementary_Data

## Abbreviations

BBB: blood–brain barrier
BPND: non-displaceable binding potential
CADASIL: cerebral autosomal dominant arteriopathy with subcortical infarcts and leukoencephalopathy
NAWM: normal-appearing white matter
SVD: cerebral small vessel disease
WMH: white matter hyperintensities
