# Supplementary material for: Microglial activation and blood–brain barrier permeability in cerebral small vessel disease
Source: Brain. 2021 May 17;144(5):1361–71. doi: 10.1093/brain/awab003 (PMC8874873; doi:10.1093/brain/awab003)
Supplement: awab003_Supplementary_Data [file awab003_Supplementary_Data.pdf]

Supplementary Material

Appendix 1: Cognition

Table A1: Z scores for the various cognitive domains for control, sporadic SVD and CADASIL groups. P values for comparisons between the sporadic SVD and control groups, and the CADASIL and control groups are shown. Analyses were controlled for age, sex and premorbid IQ. Significant p values are shown in bold.

Cognitive index	Group (Mean (SD))			P value	P value
	Control N=20	Sporadic SVD N=20	CADASIL N=20	SVD vs. control	CADASIL vs. control
**GC**	0.22 (0.52)	-0.16 (0.80)	-0.37 (0.96)	**0.021**	**0.011**
**EF**	0.15 (0.87)	-0.28 (1.09)	-0.20 (1.44)	0.128	0.649
**PS**	0.42 (0.43)	-0.58 (1.12)	-0.61 (1.85)	**<0.001**	**0.027**
**WoM**	-0.01 (0.76)	-0.09 (0.95)	-0.36 (0.61)	1.000	0.099
**LTM**	0.30 (0.75)	0.30 (0.71)	-0.32 (0.78)	1.000	**0.007**

Table A2: Group comparisons of MRI parameters between sporadic SVD and control, and CADASIL and control. Significant p values are shown in bold. WMH and lacune volume are expressed as raw values and then normalised to intracranial volume, and expressed as a % of total intracranial volume.

MRI parameter		Group (Median [Q1, Q3])			P value	
		Control	Sporadic SVD	CADASIL	SVD vs. control	CADASIL vs. control
**WMH volume**	**Raw (cm^3)**	0.98 [0.37, 2.560]	15.51 [11.17, 33.69]	50.65 [40.40, 87.26]		
	**Normalised (%)**	0.05 [0.02, 0.19]	0.97 [0.66, 2.01]	3.06 [2.26, 4.85]	**<0.001**	**<0.001**
**Lacune volume**	**Raw (cm^3)**	0.00 [0.00, 0.00]	0.140 [0.04, 2.30]	0.210 [0.00, 1.66]		
	**Normalised (%)**	0.00 [0.00, 0.00]	0.01 [0.00, 0.01]	0.01 [0.00, 0.10]	**<0.001**	**<0.001**
**Lacune count**		0.00 [0.00, 0.00]	1.00 [0.75, 2.25]	2.00 [0.00, 8.50]	**<0.001**	**<0.001**
**CMB count**		0.00 [0.00, 0.00]	1.00 [0.00, 2.50]	0.00 [0.00, 5.50]	**0.002**	**0.008**

Appendix 2: Blood Biomarkers

Principal component analysis

Details on the variance explained by each of the dimensions and contributions of the biomarkers for the sporadic SVD group (Figure A2) and CADASIL group (Figure A3).

A

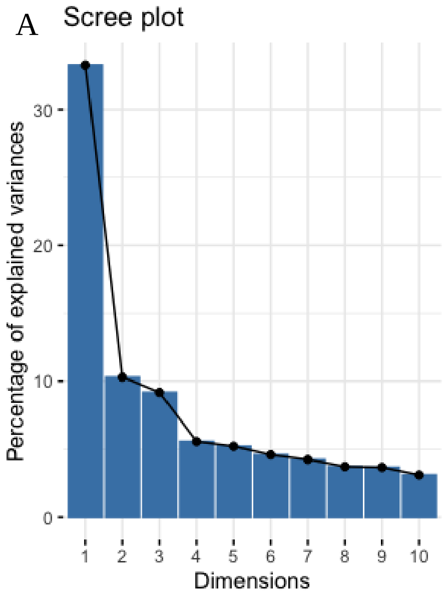

C

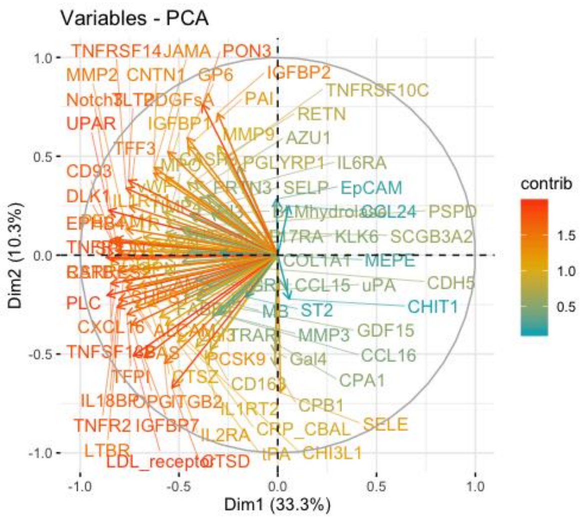

B

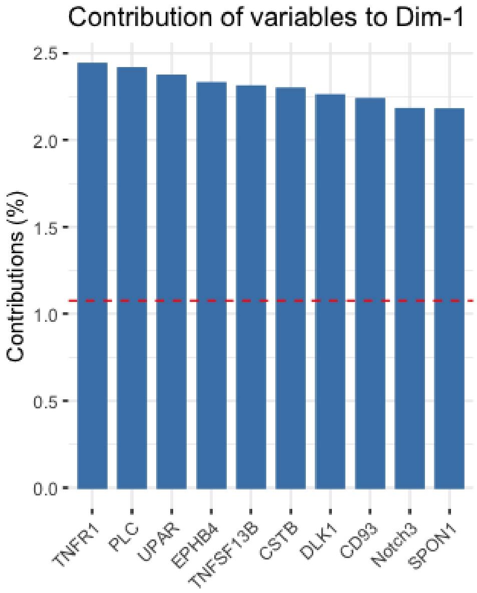

Figure A1: Principal component analysis (PCA) for the blood biomarkers in the sporadic SVD group. A) Variance explained by each of the dimensions. B) Contributions of the top 10 biomarkers to dimension 1. C) Graph of the variables for dimensions 1 and 2.

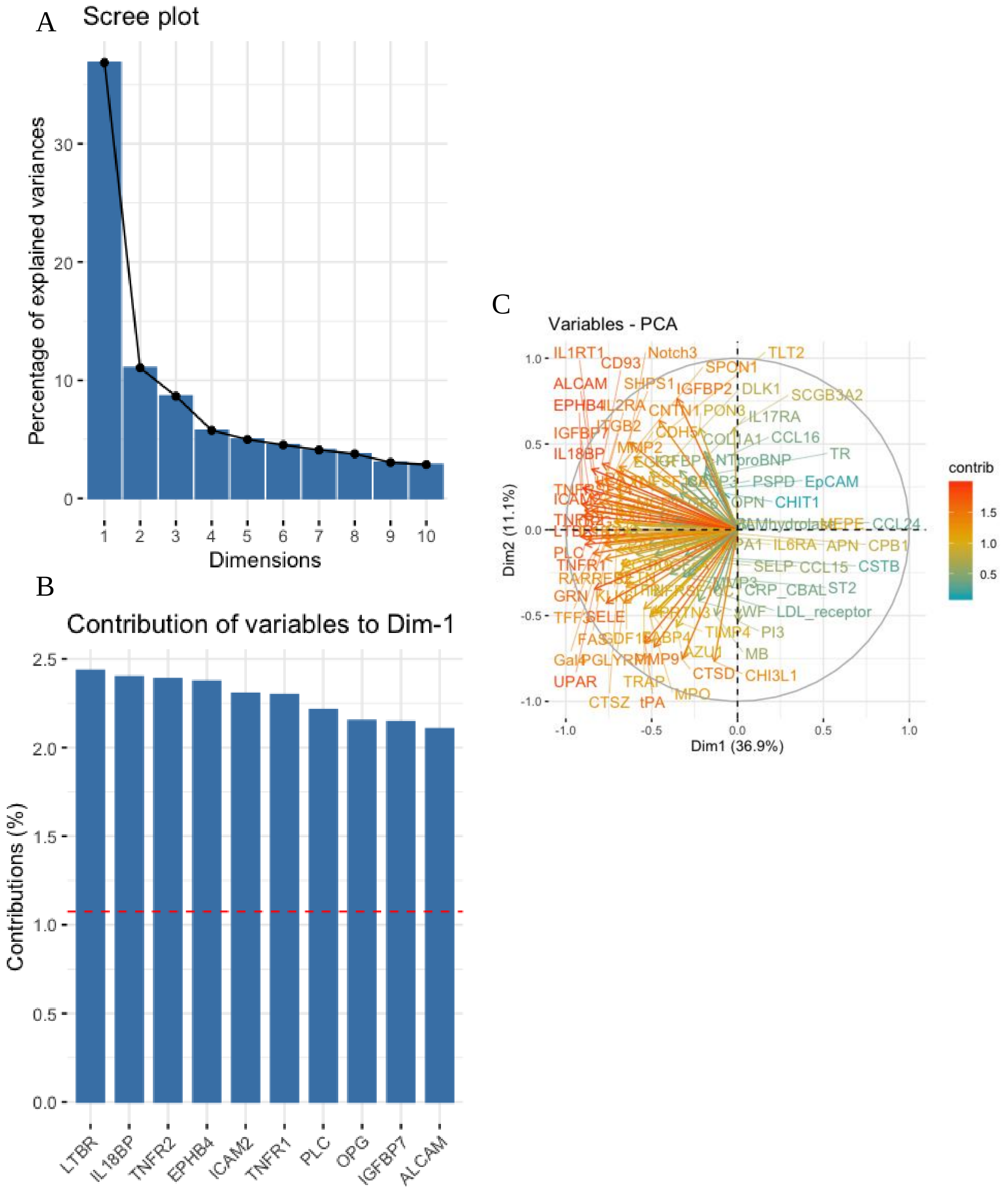

Figure A2: Principal component analysis (PCA) for the blood biomarkers in the CADASIL group. A) Variance explained by each of the dimensions. B) Contributions

of the top 10 biomarkers to dimension 1. C) Graph of the variables for dimensions 1 and 2.
